# Tell me *why*: the missing *w* in episodic memory’s *what*, *where*, and *when*

**DOI:** 10.3758/s13415-024-01234-4

**Published:** 2024-10-25

**Authors:** Fernanda Morales-Calva, Stephanie L. Leal

**Affiliations:** 1 Department of Psychological Sciences, Rice University, Houston, TX USA; 2 Department of Integrative Biology & Physiology, UCLA, 621 Charles E Young Dr S, Los Angeles, CA 90095 USA

**Keywords:** Episodic memory, Individual differences, Emotion, Hippocampus, Amygdala, Prefrontal cortex

## Abstract

Endel Tulving defined episodic memory as consisting of a spatiotemporal context. It enables us to recollect personal experiences of people, things, places, and situations. In other words, it is made up of *what*, *where*, and *when* components. However, this definition does not include arguably the most important aspect of episodic memory: the *why*. Understanding why we remember has important implications to better understand how our memory system works and as a potential target of intervention for memory impairment. The intrinsic and extrinsic factors related to why some experiences are better remembered than others have been widely investigated but largely independently studied. How these factors interact with one another to drive an event to become a lasting memory is still unknown. This review summarizes research examining the *why* of episodic memory, where we aim to uncover the factors that drive core features of our memory. We discuss the concept of episodic memory examining the *what*, *where*, and *when*, and how the *why* is essential to each of these key components of episodic memory. Furthermore, we discuss the neural mechanisms known to support our rich episodic memories and how a w*hy* signal may provide critical modulatory impact on neural activity and communication. Finally, we discuss the individual differences that may further drive why we remember certain experiences over others. A better understanding of these elements, and how we experience memory in daily life, can elucidate why we remember what we remember, providing important insight into the overarching goal of our memory system.

## Episodic memory: remembering the *what*, *where*, and *when*

Our memories make us who we are. We are continuously learning to adapt to a world that is always changing, and our memory for past events allows us to interact meaningfully with the world around us. Our ability to store and retrieve information about unique personal events, known as *episodic memory*, allows us to reflect upon our past and plan for our future, which is critical for everyday functioning (Dickerson & Eichenbaum, 2010). Endel Tulving first coined the term episodic memory to describe a long-term memory system that receives and stores information about temporally dated episodes or events, and temporal-spatial relations among these events (Tulving, 1972). Episodic memory can be conceptualized as a conscious and intentional recollection of personal experiences that consists of a spatiotemporal context (Schacter, 1987). Thus, episodic memory allows us to remember the lyrics to that catchy Backstreet Boys song, as well as recollections of people, things, and situations, which is why it is sometimes referred to as mental time travel. The key features of episodic memory have been defined as including *what*, *where*, and *when* components.

Much of the seminal work in animal research has investigated the *where*, or the spatial aspects of memory, in which this domain has been primarily studied through exploration and navigation patterns, and place and grid cell activation studies (Bush et al., 2015; Moser et al., 2015; Tolman, 1948). On the other hand, human studies have often focused on the *what*, or the content of memory, by exploring how people remember words, objects, pictures, etc. (J.B.Brewer et al., 1998; Davachi, 2006; O’Kane et al., 2005). The least-studied domain thus far, although interest in this domain has greatly increased in recent years, is the investigation of the *when* component, or the temporal aspects of memory (Eichenbaum, 2013, 2017a; Rubin & Umanath, 2015). We argue that across all of these domains, an important unifying  *W* is missing from our characterization of episodic memory: the *why* domain. We propose that the *why* provides the *context* in which these three well-studied components (*what, when, where*) of episodic memory are rooted, indicating the significance or relevance of an event, which is heavily dependent on the individual and their past experiences. The *why* domain of episodic memory provides the reasons and motivations underlying why certain events are remembered. Two books have recently been published on this very topic (Madan, 2024; Ranganath, 2024), highlighting the importance of thinking about the *why* of memory. It involves examining the emotional significance, personal relevance, and contextual connections that contribute to the retention and forgetting of our experiences.

Moreover, we maintain that the *why* is essential for understanding how our memory system works. The *why* of an episodic memory may be tagged in multiple ways—repetition, novelty, emotion, attention, reward, etc.—and is a core feature of episodic memory. Our brains must prioritize what information gets remembered and forgotten, and the *why* provides essential context in which to make this determination. In fact, animal models primarily rely on motivation and reward (i.e., making learning meaningful) to measure whether learning is taking place (Tolman & Honzik, 1930). If the *why* component is not included, we may not be able to observe whether learning is taking place. Similarly, human studies provide compensation to provide motivation for participating in a study. However, more importantly, there are intrinsic factors that drive what we remember and forget that heavily rely on the context of the experience and what we might use that information for in the future. After all, the purpose of memory is to remember the past to shape future behavior.

## Autobiographical memory

Autobiographical memory (AM), or memory of past experiences that were personally significant or relevant, plays a pivotal role in shaping our personal histories and sense of self (W.F. Brewer, 1986). Autobiographical memories provide a broader context and continuity to one’s life story as they help build life narratives that connect our past to our present and to our (imagined) future (Hutmacher & Morgenroth, 2022). Thus, AM is an important area of research that ties well into the *why* of episodic memory given that individual and personal experiences can greatly impact why we remember *what*, *where*, and *when*. One way that AM has been explored is within three main contexts: directive, self, and social (Sow et al., 2023). The directive function describes the use of past experiences to direct or guide current and future actions, thoughts, and behaviors (Vranić et al., 2018), where autobiographical information supports decision-making by retrieving past experiences to enable solving of current problems and predict future events (Bluck et al., 2005). The self function refers to the use of personal information to maintain a sense of self (Vranić et al., 2018). This sense of identity is then conveyed to others through the expression and sharing of AMs (Conway & Pleydell-Pearce, 2000). Finally, the social function refers to social bonding via retrieving and sharing personal memories, where AMs are viewed as vignettes used to initiate, develop, foster, and maintain social bonds and relations (Bluck & Habermas, 2001; Sow et al., 2023).

The retrieval of AMs is characterized by the association of various details related to the remembered event, encompassing both conceptualized and perceptual episodic elements (Sheldon et al., 2019). Autobiographical memory is also an important component when thinking about what we recollect, as AM is crucial for shaping individual identity and emotional experience (Conway & Pleydell-Pearce, 2000). Autobiographical memories are posited to be transient mental constructs influenced by the goals of the working self. This interaction shapes the cues that activate specific memories, forming a coherent narrative of personal experiences aligned with current goals and motivations. This model integrates cognitive, emotional, and personal dimensions, offering a comprehensive framework for understanding what we remember. Autobiographical memories are not static representations, but are dynamically constructed based on retrieval goals and context. Thus, the evolution of our memory systems may have supported AM, which has promoted survivorship (Sow et al., 2023). Autobiographical memory is an important feature of memory to consider when thinking about the *why* of episodic memory, given the individualized links and significance of the remembered event.

## The *why* of episodic memory

Whereas extensive research has focused on comprehending the *what*, *where*, and *when* components of episodic memory, understanding the underlying reasons for *why* we remember certain events remains a complex challenge for cognitive and behavioral neuroscience. In the current review, we explore the multifaceted aspects of episodic memory that contribute to the formation and retrieval of memories. By starting to unravel the enigma of *why* we remember, this review will shed light on the intricate interplay of factors in the formation and retrieval of episodic memories. We speculate that the *why* is part of deeper cognitive and adaptative functions of episodic memory that more closely mimic real-world experiences yet are more difficult to examine in well-controlled laboratory settings, binding together the other three *W*s. This trade-off presents a major challenge toward understanding real-world episodic memory. Uncovering how our memory system selects what to remember and what to forget is essential to understanding how our memory system works in a complex and dynamic world, and determining ways in which we may be able to enhance and rehabilitate it in the face of deficits. This has crucial implications for fields ranging from psychology and neuroscience to marketing, education, and clinical interventions. Moreover, this knowledge can help us uncover the evolutionary significance of episodic memory. Overall, including *why* in our study of episodic memory will provide a more holistic understanding of the purpose of memory and how the brain can process multimodal information. We propose that the *why* serves as the glue linking the content and context of our memories.

To better understand and characterize why we remember, we will explore the three known *W*s of episodic memory through neurobiology and behavior while identifying elements in which the *why* may aid or drive the encoding and recollection of events. We will expand on some possible drivers of *why* we remember and how these connect, contextualize, and ground the *what, where,* and *when*. Finally, we will discuss important elements to explore when considering understanding the *why* in episodic memory frameworks.

## Neurobiology of *what, where, when,* and *why*

**Fig. 1:**
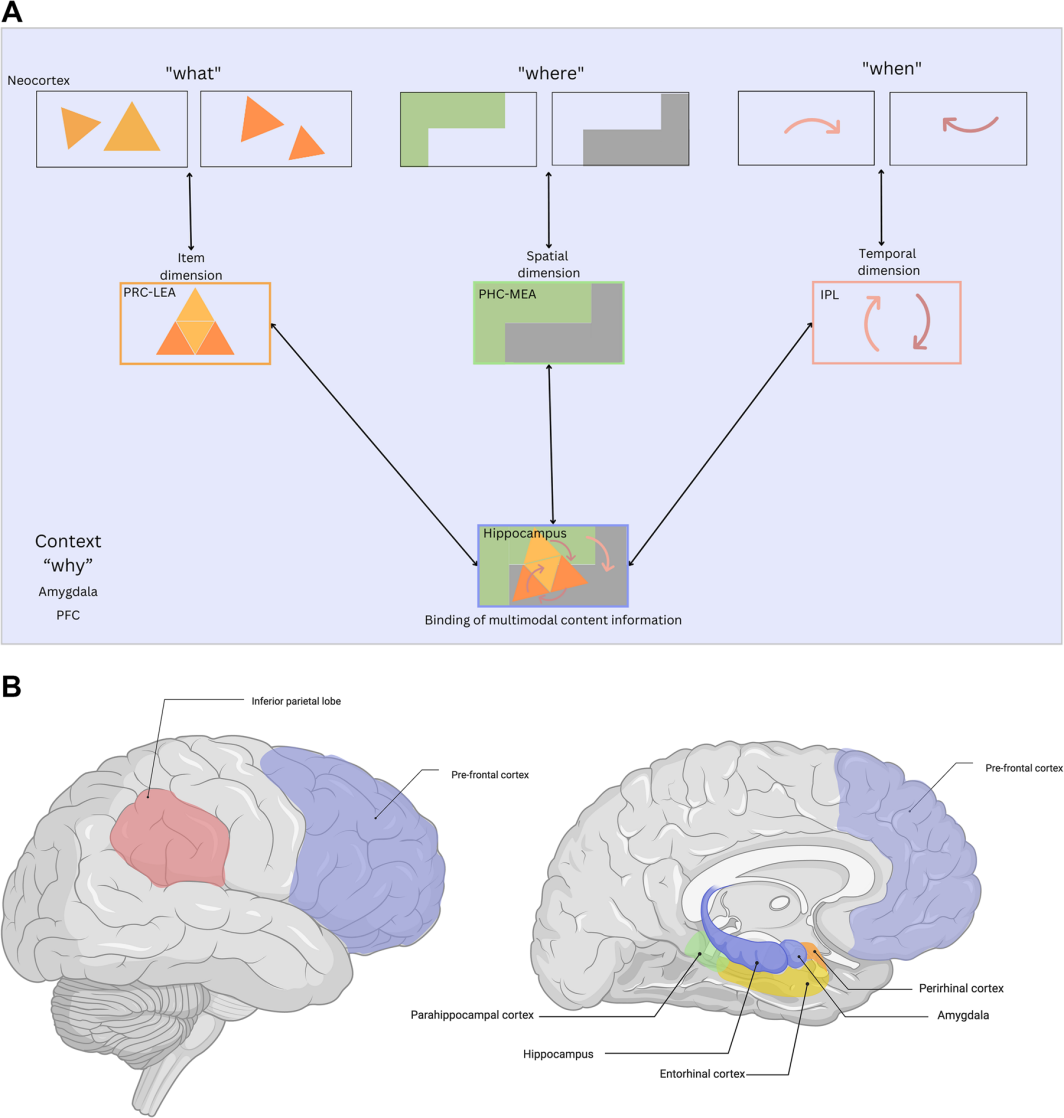
**Holistic view of hierarchical processing in episodic memory. A**) Proposed hierarchy of episodic memory content processing from neocortex to hippocampus, incorporating the *why* as context. Adapted and expanded from Eichenbaum et al. (2007). ***B***) Brain regions known to be involved in the processing of *what, where, when,* and *why*. Graphic B created in BioRender. Morales-Calva, (2024). BioRender.com/x17t926

The neurobiological underpinnings of episodic memory are primarily found within the medial temporal lobe (MTL), which includes the hippocampus, amygdala, and surrounding entorhinal (EC), parahippocampal (PHC), and perirhinal (PRC) cortices (Baxter et al., 2011). The organization of the MTL suggests a hierarchical processing format, where encoded information is processed from the neocortex to the PHC and PRC, then to the medial and lateral EC, respectively, and ultimately reaches the hippocampus, where multimodal information is processed (Dickerson & Eichenbaum, 2010; Raslau et al., 2015; Squire et al., 2004). The PRC primarily receives information about the qualities of stored items in an event from the ventral, or *what*, stream, whereas the PHC receives spatial information from the dorsal, or *where*, stream (Raslau et al., 2015; Yonelinas et al., 2019). The presence of a *when* pathway has been less studied; however, research has shown the inferior parietal lobe (IPL) to be involved in the processing of temporal information (Battelli et al., 2007) during encoding. These streams have previously been theorized to converge into the hippocampus, which processes multimodal incoming information by taking these inputs and binding the representations across *what, where,* and *when* domains (Fig. 1) (Dickerson & Eichenbaum, 2010). During an event, all areas in the MTL are interconnected and receive unimodal and multimodal information from the senses and association areas (Lavenex & Amaral, 2000). When it comes to the retrieval of memories, even though the hippocampus and surrounding cortical regions play a key role in episodic memory, it is not entirely accomplished within the MTL. Rather, a widespread network encompassing frontal and parietal cortices is also necessary for memory retrieval (Chini & Hanganu-Opatz, 2021; Kirchhoff et al., 2000; Rugg, 2022).

We propose that the *why* of our memories shapes what we remember and may be aided by the high interconnectivity of the regions within the MTL (i.e., binding and associations processed in the hippocampus) as well as to other brain areas. For example, the amygdala and its connections with the hippocampus and cortical regions, such as the prefrontal cortex (PFC), work together to modulate the processing of episodic memories (Zheng et al., 2017). The amygdala modulates hippocampal function via neurotransmitters, such as norepinephrine (Gallagher et al., 1977; Phelps, 2004). In particular, the connections between the basolateral amygdala (BLA) and the hippocampus are thought to modulate the strength of a memory (McGaugh, 2004). Although the amygdala is often left out when defining the MTL, it is an essential region and plays an important role in shaping the *why* of memory by signaling biologically relevant events (i.e., it is not simply a “fear center”) (Markowitsch & Staniloiu, 2011; Pessoa & Adolphs, 2010; Sander et al., 2003). The frequency of this theoretical disconnection highlights that certain brain regions that play a pivotal role in memory are often overlooked when conceptualizing episodic memory, and this disconnection has extended into understanding clinical conditions, such as Alzheimer’s disease (AD) (Stouffer et al., 2024). Nonetheless, the entire brain network associated with memory is important in providing a rich context in which to evaluate episodic memory.

The PFC is known to support control of memory processing, such as permitting the retrieval of context-appropriate memories and suppressing competing memories (Gilboa, 2004; Preston & Eichenbaum, 2013), where the PFC and hippocampus interact bidirectionally (Eichenbaum, 2017b). This highlights the value of exploring whole-brain interactions and connectivity in the study of episodic memory retrieval, as its processing relies on more than the MTL and often aids in more contextual components of episodic memory (e.g., biological significance, cognitive control). We propose that the exploration of the role these regions and how they interact with the core features of episodic memory can aid in understanding and conceptualizing the *why*.

Tulving (2002) originally proposed episodic memory to be unique to humans. The storage and retrieval of event-related memories have served as an evolutionary advantage, such as making predictions and planning for the future based on spatially and temporally specific information about experiences (Klein et al., 2010). Episodic memory allows for quick adaptation to changing circumstances based on stored information. However, the core properties of episodic memory are present across all mammals as well as a number of bird species, with the hippocampus being shared as a homologous structure across species to allow for the mechanisms of episodic memory formation (Allen & Fortin, 2013; Clayton & Russell, 2009; Mendl & Paul, 2008). In a study by Ergorul and Eichenbaum (2004), rats were tested on their episodic-like memory, where they sampled odors (*what*), in unique places (*where*), and tested their memory for the order in which these events happened (*when*) by presenting a pair of odors in their original locations. Rats used a combination of *where* and *what* information to judge *when* these events occurred; however, rats with hippocampal damage could not effectively combine these three qualities of the experience, highlighting the importance of the hippocampus in binding across these domains. This demonstrated that the components of episodic memory were not unique to humans (Salwiczek et al., 2008). This also highlights the opportunity to examine various aspects of the *why* of episodic memory across species and allows for different levels of analysis to be conducted within this space (Marr, 1971).

While episodic memory is not unique to humans, one aspect of episodic memory thought to be specific to humans is *mental time travel* (Suddendorf & Corballis, 1997). Mental time travel refers to not only the ability to reconstruct personal events from the *past* but to be able to imagine possible scenarios in the *future*, also known as episodic foresight (Miloyan et al., 2019). While this has been exclusively examined in humans, some argue that mental time travel is inaccessible to study in animals beyond humans, making it difficult to determine whether mental time travel is truly human-specific (Mendl & Paul, 2008). Nevertheless, cross-species studies offer valuable insights into the fundamental mechanisms underlying all domains of episodic memory, shedding light on the commonalities and unique adaptations across organisms. Moreover, examining memory formation and function across species allows researchers to employ a variety of experimental techniques, ranging from behavioral observations to detailed *in vivo* and *postmortem* neurobiological investigations, thus facilitating a more nuanced exploration of memory at various levels of analysis (e.g., computational, algorithmic, implementational).

## *What* do we remember? The item domain of remembered events

The *what* component of episodic memory refers to the content and features of events that are recalled. The *what* includes sensory and perceptual information of an episode, ranging across modalities and often includes associations between remembered items. The *what* domain has been examined across species and focuses on the retrieval of previously encountered stimuli and our ability to recognize them as such. Recognition can be conceptualized as two distinct memory processes: *recollection and familiarity* (Henson et al., 1999). Recollection refers to the retrieval of information about a specific episode, whereas familiarity reflects a more global measure of the strength of the memory (Westerberg et al., 2006; Yonelinas et al., 2010). There is some debate regarding how the MTL contributes to familiarity and recollection. Some research finds that different MTL regions are equally important for both processes (Brown & Aggleton, 2001; Rugg & Yonelinas, 2003), whereas others suggest that specific and distinct MTL regions contribute to each particular retrieval process—specifically the hippocampus being essential for recollection and surrounding MTL cortices for familiarity (Manns et al., 2003; Wixted & Squire, 2004). Historically, different tasks have been used to measure recollection (Yes/No paradigm—items presented one at a time) (Aggleton, 1985; Bastin & Van der Linden, 2003; Bayley et al., 2008) and familiarity (Forced-choice paradigm—multiple items presented at a time) independently (Medin & Schaffer, 1978; Mishkin & Delacour, 1975; Nosofsky & Zaki, 2003). While these concepts are helpful in characterizing *what* we remember, these models alone may not be enough to explain memory in the real-world that is complicated by interference across our memories.

The interference across experiences in which items may share features can impact one’s ability to recognize an item (Reagh & Yassa, 2014a). Everyday experiences are full of interference, especially when certain elements are repeated across time. Computational models propose that the hippocampus can perform two key computations to reduce interference across our experiences: *pattern separation and pattern completion*. It is the balance between the two computations that permit us to have rich episodic memories (Leal & Yassa, 2015). Pattern separation is the ability to discriminate among similar experiences and allows them to be stored as distinct representations (Yassa & Stark, 2011). For example, differentiating between two parties you attended within the last month, or remembering where you put your keys today versus yesterday. This process relies on the dentate gyrus (DG) of the hippocampus and its mossy fiber connections to CA3 (Larocque et al., 2013). The sparse firing within the DG allows it to code unique experiences; therefore, the DG is known as the universal pattern separator. Pattern completion is the ability to generalize when given partial sensory cues and makes recognition dependable in the face of noise (Yassa & Stark, 2011). For example, being able to recognize your friend after they got a haircut or a new pair of glasses, even though they may look a bit different. Pattern completion relies on the CA3 subfield of the hippocampus, where its recurrent collaterals form a positive feedback loop, synapsing largely onto itself (Leal & Yassa, 2015; Leal et al., 2019). To tax pattern separation in humans, mnemonic discrimination tasks have been developed, which include both repeated items and similar lure items during a memory test. It is the inclusion of similar items that make it highly sensitive to hippocampal function by placing strong demands on pattern separation (i.e., greater interference) (Stark et al., 2019).

The ability to remember items is an essential feature of episodic memory. Nonetheless, why we remember certain items and how this drives memory processing from the moment events are encoded to when we retrieve them requires understanding the context in which the event occurred. Context here does not refer to physical space, but various features contributing to the *why*. Exploring the interplay between the perceptual and conceptual details of an event allows for a more nuanced understanding of why we remember what we remember.

### Memorability

A growing body of work aims to understand what factors drive certain items to be better remembered than others. *Memorability* refers to the systematic variation with which some events are better remembered than others (Rust & Mehrpour, 2020). This is an intrinsic property of items (including faces, words, images, voices, and movements) that is shared and highly consistent across subjects (Madan, 2021); it is said to be time, context, and coding type independent (Goetschalckx et al., 2017, 2019). Memorability has also been shown independent of attention and priming effects (Bainbridge, 2020), or top-down influences, as the phenomenon is considered to be “automatic” (Grande et al., 2021). This is highlighted by the fact that many of the factors that determine memorability are not intuitive; it turns out that humans are bad at predicting how memorable a stimulus can be (Rust & Mehrpour, 2020), where we tend to predict that aesthetic and interesting objects will be more memorable, but this is only weakly correlated with actual memorability scores based on memory performance (Khosla et al., 2015).

Images containing people and man-made elements are highly memorable, in contrast to images of nature scenes, which are more forgettable (Goetschalckx & Wagemans, 2019). Pictures with atypical content are usually better remembered, as are pictures with higher emotional arousal or valence (Khosla et al., 2015; Morales-Calva & Leal, 2024), and images that evoke disgust, amusement, or fear tend to be more memorable than images that evoke contentment (Bainbridge et al., 2013). These factors are also largely applicable to pictures of faces, where atypical faces are more memorable than more typical-looking faces. In addition, images that are brighter, more colorful, larger, well-centered, and uncluttered tend to be more memorable (Goetschalckx et al., 2019), extending beyond experimental stimuli, with even certain artwork being more memorable than other pieces (Davis & Bainbridge, 2023).

The currently known factors that influence memorability can account for 50% to 75% of the variance in memory performance (Rust & Mehrpour, 2020), suggesting that memorability, or *what* is better remembered, could help us to understand further *why* some elements are better remembered. It is important to note that there is still a sizable percentage of variation that cannot be explained purely by memorability, and whereas several attributes for an image have shown correlations with memorability, no singular attribute has been found that can function as a proxy for it. It is also important to consider how memorability is measured and in what context. For example, something that is deemed memorable may be memorable for the central aspects, or gist, of an experience, while the details may be more forgotten (Morales-Calva & Leal, 2024). On the other hand, individual differences might also influence what drives elements to be more memorable, ideas that we will discuss in more detail in a later section. Thus, it is important to consider why this phenomenon occurs and how this impacts why we remember certain elements of an experience better than others.

### Emotional significance

Emotion plays an integral role in memory formation. Previous work has shown that the emotional valence and arousal of an experience can influence remembering and forgetting (McGaugh, 2004) and that our brains prioritize emotional information to consolidate after sleep (Denis et al., 2022; Payne et al., 2008). While we often consider emotional experiences to be better remembered, *gist versus detail trade-offs* are known to occur, where the central aspect of a memory, or the gist, is better remembered while the details are more often forgotten (Kensinger, 2009; Leal et al., 2014; Loftus et al., 1987; Mather & Sutherland, 2011). Thus, an emotional context biases what we remember, highlighting a key role of the *why* in episodic memory shaping *what* we remember.

Stress hormones released by the adrenal gland during an emotional experience also play a vital role in the effects of emotional arousal on making lasting memories (McGaugh, 2004). The amygdala is thought to be crucial in the processing of emotional memories through its influence and modulation of hippocampal representations (Zheng et al., 2017). Thus, whereas the hippocampus plays a key role in processing key features of the episodic memory (e.g., *what, where, when*), the amygdala can enhance or impair memory by signaling their emotional significance (e.g., *why*) (McGaugh, 2013; Phelps, 2004). Even though the amygdala and hippocampus can operate independently, they exhibit mutual dependence when coding contextual information about the experience, which is necessary for our brains to prioritize which information should be remembered long-term.

### Attention and repetition

Attention is a fundamental cognitive process crucial for understanding what we remember. Attention acts as a filter, determining which information gets encoded in the first place (Siegel & Castel, 2018). Without attention, our perception would be inundated with an overwhelming number of stimuli, making it impossible to discern the relevant details to remember (Adam & Vogel, 2016). Through the use of selective attention, we are able to focus on specific or relevant elements in our environment, allowing us to encode and retain pertinent information (Schupp et al., 2007). Moreover, attention has been shown to help stabilize hippocampal representations in promoting episodic encoding (Aly & Turk-Browne, 2016; deBettencourt et al., 2015).

Another factor that is relevant to *why* is repetition. Repetition is often thought to enhance memory, as we often do when studying for an exam or trying to remember a phone number; however, the opposite may as well occur, in which repetition may lead to poorer memory. One possibility is that repetition of previous experiences can facilitate memory by reducing the attentional resources needed for its cognitive processing; where repetition can support the reinforcement of neural pathways associated with particular events as well as in the strengthening of connections between existing information, fostering familiarity/generalization, and facilitating the retention of information for the long term (Tulving & Schacter, 1990). Alternatively, repetition has been proposed to negatively affect memory processing by increasing interference between novel and existing representations (Kim et al., 2012). Also, mnemonic discrimination paradigms have shown that stimulus repetition during encoding benefits target recognition but impairs lure discrimination (Reagh & Yassa, 2014a). Hence, factors, such as attention and repetition, provide important contextual information about a given experience. Where certain features of an event may grab your attention, because they are important and relevant to the situation, and if experiences or items are repeated in our environment, this may bias how that information gets processed and stored.

## *Where *do we remember? The spatial domain of remembered events

One of the main features of episodic memory is its capacity to encode and retrieve spatial information associated with events. Spatial memory refers to memory for dimensional configurations and the ability to navigate a familiar or new environment (Fan et al., 2023). We are able to develop a cognitive map, which becomes more detailed over time, to acquire, code, store, and recall information about the locations in our everyday environment (Ekstrom et al., 2018). Previous studies have examined the *where* pathway via the PHC to the hippocampus (Buffalo, 2015; Connor & Knierim, 2017; Strange et al., 2014). These regions play a pivotal role in forming cognitive maps (Tolman, 1948), allowing us to remember the *where* information. Place cells are found in the hippocampus and fire when a rodent is in one specific location (O’Keefe & Dostrovsky, 1971), whereas grid cells are found in the entorhinal cortex and create a map of the environment (Moser et al., 2015). Studies in humans have also shown an important role for the hippocampus in memory-guided spatial navigation. Patients with hippocampal lesions show more reliance on multisensory input for successful spatial memory performance (Iggena et al., 2023). This is also true for individual objects displaced by varying degrees; older adults showed a deficit in memory performance compared with older adults (Reagh et al., 2014). Additionally, performance on this task has shown consistent neuroimaging activation in the PHC and MEC (Reagh & Yassa, 2014b), the regions associated with spatial information in animal models.

It is important to note that there is existing debate on whether spatial and episodic memory are different systems or intrinsically interconnected (Fan et al., 2023; Theves et al., 2020). We argue that space is one *content* element that comprises our episodic memories and is not unique in the sense that for certain goals *where* information may be most important and relevant to remember, whereas for other goals, *what* or *when* information may be most relevant. Perhaps it is the integration or association between these elements that gives rise to our rich episodic memories. Again, we see the *why* emerge as essential to determine which aspects of memory are most significant for our goals.

### Rewards and goals

The spatial component of episodic memory holds evolutionary significance, reflecting our need to navigate and survive in our environment (Meade et al., 2019). The ability to remember where significant events occurred, such as the location of water and food sources, or potential threats, confer a distinct adaptative advantage (Becker, 2022). Hence, the *where* of episodic memory can be viewed as a product of natural selection tuned to enhance survival. A seminal study conducted by Tolman and Honzik (1930) further highlights the importance of considering the *why* in measuring *where* memory. In this study, three groups of food-deprived rats were examined in their navigation of a T maze. One group received a food reward at the end of the maze from the first day of the study, in which learning (i.e., measured by time to traverse the maze to the goal box) improved over time. The second group never found food at the end of the maze and showed little improvement, because getting out of the maze was a small reward. The third group found no food for the first 10 days, but food was presented on the 11th day. Importantly, this third group caught up to the rewarded group and even performed more quickly to receive the food reward after it became available. This experiment provides an example of *latent learning*, in which rats in the third group were learning about their environment the entire time but had no reason to show they were until that learning became relevant (i.e., to receive a food reward). This highlights the importance of the relevance of an experience in how we can measure memory.

### Novelty, familiarity, and real-world navigation

Beyond its evolutionary roots, the spatial aspect of episodic memory has practical implications for our daily lives. From recalling the location of where we parked our car to navigating through familiar and novel spaces, the *where* of memory contributes to our cognitive ecology (Hutchins, 2010). The duration of exploration patterns in rats can be indicative of novelty or recollection. This is true even when the context, not the stimulus, changes, where a previously encountered object in a novel environment is treated as novel (Panoz-Brown et al., 2016). It has been shown that rats with hippocampal damage explore novel and repeated stimuli equally, which is also the case with changing contexts (Poulter et al., 2020).

With the advancement of virtual reality technology (Seton et al., 2023) and wearable recording devices (Topalovic et al., 2023), the study of human spatial navigation inside and outside of laboratory settings is becoming more commonplace (Stangl et al., 2023). In one such study, participants completed an ambulatory spatial memory task in a virtual reality environment. Researchers found MTL theta activity to be modulated by behavioral goals, either by successful memory retrieval or spatial positions within the environment, demonstrating that the MTL can represent both memory and space during freely moving navigation (Maoz et al., 2023). The *where* component of episodic memory extends beyond static locations and spatial navigation. The ability to remember routes and spatial relationships facilitates efficient navigation in both virtual and physical spaces (Meade et al., 2019). From the evolutionary roots that shape this aspect of memory, to the intricate neural processes involved in spatial navigation, understanding the *where* enhances our understanding of the broader functions and adaptative significance of episodic memory. As technology continues to advance and our knowledge of the brain deepens, further investigations of the spatial dimension of episodic memory in the context of why holds promise for theoretical insights and practical applications in (literal and theoretical) navigation of our everyday lives.

## *When* do we remember? The temporal domain of remembered events

Our ability to recollect events from our past is characterized not only by the *what* and *where*, but also by the *when*. Our daily lives consist of a continuous stream of information; the *when* involves the temporal encoding of events, encompassing the sequence as well as the duration of experiences. The recollection of recent memories relies more heavily on episodic memory, whereas remote memories may become more semantic (Tomadesso et al., 2015). Temporal memory allows humans to navigate season changes, anticipate environmental patterns, and synchronize activities crucial for survival and reproduction. Moreover, temporal memory allows us to remember past experiences as cohesive events (Shapiro, 2019). This has clear implications in our everyday lives as our ability to project ourselves into the future relies on the integration of temporal information from past experiences. From remembering appointment times to scheduling future events, temporal memory impacts our ability to plan, anticipate, and navigate life (Klein et al., 2010). Integration of temporal details is essential for decision-making, goal-setting, and the construction of future scenarios. This has important implications for time management and future planning and in fields such as education and interventions for people with temporal memory deficits.

Previous studies have shown the MTL is involved in temporal processing (Benchenane et al., 2010; Preston & Eichenbaum, 2013; Tsao et al., 2018), illustrating that there are intricate neural mechanisms underlying our ability to remember when an event occurred as well as the structure of that event. Additional studies in rats have shown the anterior thalamic nuclei to be essential as part of an extended hippocampal system, which supports the encoding of temporal order of sequences (Wolff et al., 2006). One key facet of temporal memory inherently involves models of memory consolidation and how we retrieve memories after they have been encoded and how retrieval itself has the ability the change how that memory is stored.

Consolidation refers to the process of stabilizing memories and making them resistant to disruption over time (Ribot, 1881), in which memories can either decay or become resistant to disruption. Systems consolidation theory (SCT) (Squire & Alvarez, 1995), also known as the standard model of consolidation, asserts that initially, memories are in a labile state but become more stable. According to SCT, remote memories can be retrieved without the hippocampus, as neocortical connections strengthen. This standard model posits that the interaction between MTL and neocortex is only required for a limited period of time after encoding, at which point memories can be retrieved directly from cortical areas. This implies that hippocampal damage only affects recent but not remote memories, bringing the *when* of the memory to the forefront of retrieval.

However, research from amnesic patients has shown that memories do not become independent from the hippocampal system (Nadel & Moscovitcht, 1997). Multiple trace theory (MTT) challenges the ideas of the standard model by stating that the hippocampus is always required for the retrieval of episodic memories regardless of the time that has elapsed (Nadel & Moscovitcht, 1997; Nadel et al., 2000). MTT hypothesizes that every time a memory is reactivated, a new trace with the hippocampus is formed. Within this model older memories are reactivated, forming more traces, and making them more resistant to disruption. However, the temporal gradient for this model is flat, where if the hippocampus is completely damaged, both recent and remote memories, would be lost.

Competitive trace theory (CTT) (Yassa & Reagh, 2013) presents a continuum model that tries to address the role of the hippocampus in the retrieval of remote memories. According to the CTT, the hippocampus’ main role during retrieval is that of reconstructing memories by using overlapping traces. Competitive trace theory posits that as memories get older, they become more decontextualized due to competition among overlapping representations, becoming more reliant on storage within the neocortex. Based on hippocampal pattern separation and completion, CTT states that in the hippocampus, traces are non-overlapping due to pattern separation, whereas in the neocortex, overlapping representations are strengthened.

Theories of consolidation help to shed light on the importance of the temporal aspect of memory during retrieval. It is important to note that long-term memory has been considered to include varying time delays, ranging from minutes to decades, making it quite complicated to study in laboratory settings. Furthermore, the temporal structure within an event or across event boundaries is another temporal aspect of episodic memory to consider, further pointing to the vast temporal contributions to episodic memory. Understanding the temporal elements of episodic memory also helps to explain why some events are better preserved over time, whereas others are forgotten or altered.

### Forgetting

To understand why we remember certain experiences, it is essential to understand why we forget. Although counterintuitive at first, forgetting is a necessary feature of our memory system, because it can help to avoid overload and catastrophic interference, which refers to the disruption or complete elimination of prior learning resulting from new learning (Wixted, 2004; Yassa & Stark, 2011). Memories are subject to change and forgetting. Over time certain aspects of our memories become consolidated, or more stable, whereas other parts of the memory can become weaker (McGaugh, 2013). It is said that more recent memories can be more accurate but more labile, while old memories tend to be more faded but robust (Wixted, 2004). Despite the importance of *when* an event occurred, most memory tasks measure memory shortly after encoding. However, in the real-world, we more often struggle with forgetting beyond 24 h (Davis & Zhong, 2017; Leal et al., 2019; Murre & Dros, 2015). 

Forgetting can occur due to failures across different stages of memory processing, including encoding, consolidation, and retrieval failures. Forgetting can occur through decay, where with the passage of time, memories tend to “fade away”, and our ability to remember seems to decrease (García-Rueda et al., 2022). However, this depends on many additional factors, such as the content of the memory (e.g., emotional vs. neutral), how well the experience was encoded, or whether there are strong retrieval cues to aid in recalling the experience. Retrieval-induced forgetting occurs when recalling information from long-term memory can impair the long-term retention of its representations due to inhibitory processes specific to recall (Anderson et al., 2000). These can occur because of increased retention intervals (i.e., passage of time) or inadequate retrieval cues (Miller, 2021). Forgetting can also result from the execution of goal-inappropriate processes, such as attending to irrelevant stimulus features that, when encoded, are not effective for item recognition or redirecting attention from the stimulus toward irrelevant features (Wagner & Davachi, 2001). Another theory suggests that undifferentiated traces occur during encoding, where the inability to separate overlapping representations culminates in interference and forgetting (McClelland et al., 1995). Finally, it is possible that recognition does not occur due to inaccessibility to details (Guerin et al., 2012) or retroactive interference (Miller & Matzel, 2006) during retrieval processes.

Overall, forgetting allows prioritization of what we should remember, based on goals, motivations, emotional context, etc., reducing priority of extraneous information that may not be essential. Thus, a better understanding of why we forget can provide us with the answer to the question of why we remember.

### Event boundaries

Within a typical day, we experience many occurrences that seemingly flow continuously from event to event. Models of event perception postulate that we notice shifts in a context as *event boundaries* (Radvansky, 2012; Zacks et al., 2007), where we are able to take continuous sensory inputs and segment them. These boundaries help to update mental representations of contexts, which may promote the selection of behaviors suited for current environments (Clewett et al., 2019). Event boundaries can modulate how we remember the passing of time (Waldum & Sahakyan, 2013) as well as the temporal distance between events (Ezzyat & Davachi, 2014). Furthermore, it has been shown that the emotional valence of an experience can affect its perceived duration; negative events tend to be perceived as greater in duration than neutral events (Safi et al., 2024). The hippocampus has been shown to be critical for remembering the timing of events, owing to hippocampal replay being essential for the consolidation of memories for events. Replay can present during waking and sleep behavior (Karlsson & Frank, 2009). Single hippocampal neurons, termed time cells, are thought to bridge the gap between discontinuous events by encoding successive moments during temporal gaps between events, and by forming different representations when a temporal parameter is altered (MacDonald et al., 2011). These hippocampal neurons also encode events into temporally structured experiences (Eichenbaum, 2013). Our brains our wired to encode *what *and *where*, as well as features of *when* an experience occurs. Further insights into temporal memory hold promise for both theoretical and practical applications to enhance our ability to plan for the future.

## Beyond the *what*, *where*, and *when*: The *why* provides important contextual scaffolding for our memories

**Fig. 2:**
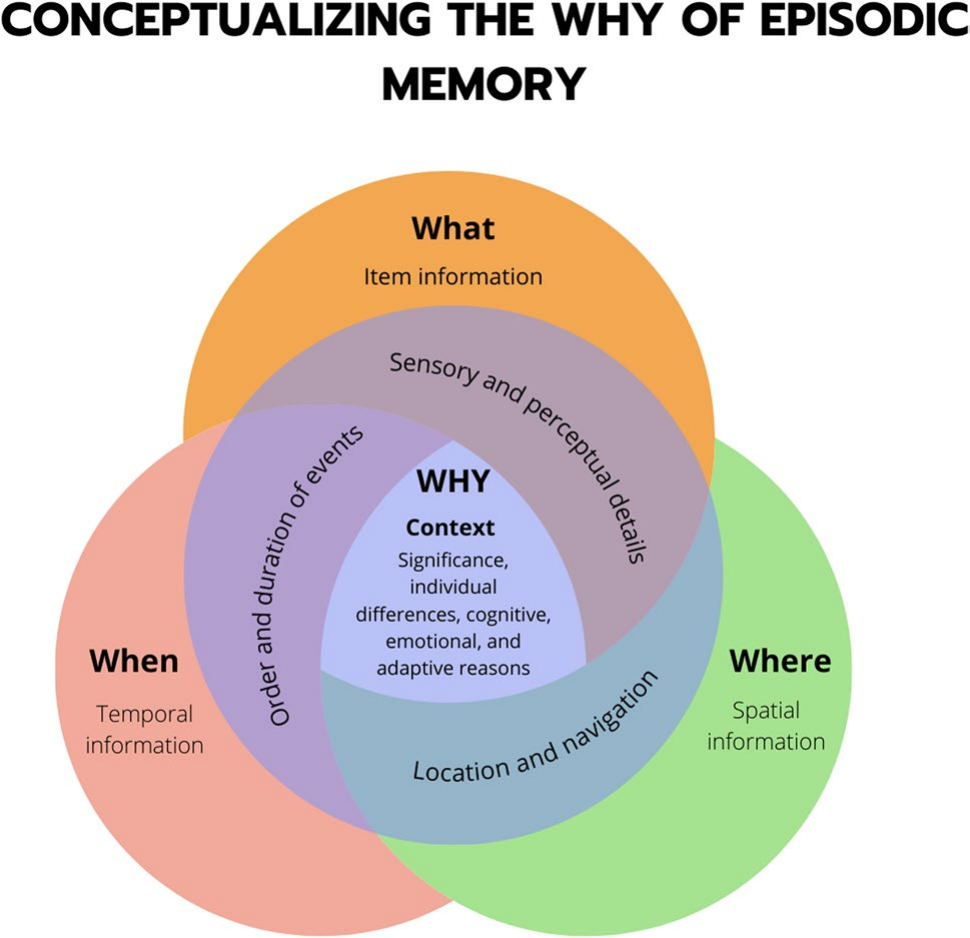
**Conceptual framework of the *why* of episodic memory.** Why we remember is central to episodic memory and binds the three core features of episodic memories: *what, where*, and *when.* We propose that remembering does not occur in a vacuum and is influenced by sensory and perceptual details of an event, the order and duration of events, and the location and navigation; witha key driver of episodic memory being the personal relevance and significance, or the context, of the event

As we have discussed, there is implicit overlap between the *what, where,* and *when* components of episodic memory, because these elements rarely occur independently of each other. In short, episodic memory involves the storage and recall of an experience that is rich in spatiotemporal context. While these features are essential components of episodic memory, they are only remembered in terms of their relevance to past, current, and future behavior. When processing an experience in present time, we must consider relevant past events and integrate them with the current experience so that we make informed decisions about the future. This fundamental trait requires consideration of the *why* component of episodic memory, in which these features may be selectively remembered depending on the *why*. Here, we posit that the binding of the three *W*s, in conjunction and interaction with individual differences is at the heart of uncovering why we remember (Fig. 2). Individual differences highlight that the *why* of memory is inherently linked to the other three *W*s and binds them together to influence memory encoding and retrieval. This is why we have discussed the *why* of episodic memory within the *what, when*, and *where* domains. However, this makes it difficult to test and isolate drivers of memory performance. While consideration of the *why* makes interpretation and study design more difficult, it is important to acknowledge this complexity and its reflection of real-world memory processing. In the following section, we will explore some of these individual elements that may be drivers or binding elements of the *why*.

### Individual differences

Individual differences that contribute to the *why* domain of episodic memory can influence how memories are formed and recollected. The *why* provides essential context for our memories. Who we are today and what we remember is molded by our past experiences, because we each hold a unique set of previous lived experiences (Wagoner, 2017). Factors, such as goals and motivations, cultural background, and age, all contribute to how we experience the world around us and drive the uniqueness of our memories. This diversity in memory formation not only reflects our individuality but also enriches our understanding of human cognition. As we explore the intricacies of episodic memory and individual differences, we gain insight into the complex interplay between our past experiences and present selves. By recognizing and embracing the varied ways in which memories are shaped, we come to better appreciate the richness and diversity of the human mind, or simply put, individual differences of lived experiences can have a profound impact on why we remember what we remember. Here, we will explore some of the many individual differences to consider in explaining why we remember.

### Internal states: motivation and mental state

Episodic memory can be influenced by internal factors as much as it can be manipulated externally. Elements, such as motivation, can affect how individuals remember an event, in which high-interest experiences that are motivating can be selectively remembered (Hu & Yang, 2023). Interest-motivated memory enhancement persists over time (McGillivray et al., 2015). Previous studies have shown that both intrinsic and extrinsic motivation can interact to influence subsequent memories (Murayama & Kuhbandner, 2011). Moreover, motivation can impact related cognitive activity, such as sustained attention and retrieval.

Other internal elements during encoding and retrieval, such as emotional and mental states, can significantly influence what we remember (Kim & Diamond, 2002). Previous studies have shown that fluctuations across emotional states affect how a memory is organized (McClay et al., 2023), as well as the strength of a memory (Mather & Sutherland, 2011). For example, stress can modulate the storage of recently acquired information. Epinephrine can influence the strength of a memory (Gold & Van Buskirk, 1975), given that events that are arousing are treated differently than non-arousing events (Cahill & Alkire, 2003). The Yerkes-Dodson law suggests that there is an optimal moderate level of arousal that enhances memory performance, while too high or too low levels of arousal can impair memory (Christianson, 1992; Yerkes & Dodson, 1908). Mood disorders, such as depression, have been associated with vulnerabilities of episodic memory (Dillon & Pizzagalli, 2018). Depression has been associated with a negativity bias (Beevers et al., 2019; Phillips, Castro et al., 2023), in which negative experiences are prioritized over positive or neutral experiences. While this bias influences what we remember, this internal state points toward *why* we remember negative experiences better. In addition, although the contribution of individual differences, such as depression, on episodic memory are well known, most basic studies of memory tend to exclude participants who report current symptoms of depression or antidepressant use. Other studies that do not exclude such samples tend not to measure or report these internal states, precluding the potential impact of these factors on the results. This likely clouds our interpretation of episodic memory findings, especially in the context of real-world experiences that inherently include these factors. Hence, it is essential to consider how these mental states may impact why we remember, given that these states can drive what our brain prioritizes, remembers, and forgets.

### Cultural influences

There are cultural differences in episodic memory performance (Leger & Gutchess, 2021). Culturally specific elements are better remembered by people who identify with that culture. Episodic memory can also differ based on several cultural factors, including how different cultures define the self. For example, Western cultures have been generally identified as more individualistic, whereas Eastern cultures as more collectivist (Lomas et al., 2023). Cross-cultural studies have shown that European and European-Americans tend to better retrieve specific events and details of recent and distant past events than their Asian and Asian-American counterparts (Wang et al., 2018). In addition, people from Eastern cultural backgrounds tend to categorize objects more holistically based on contextual relationships, whereas people from Western backgrounds tend to group objects based on underlying attributes and rules (Nisbett et al., 2001). Although it is known that different cultures process distinct aspects of information and employ diverse information-processing strategies (Boduroglu et al., 2009), the majority of published studies include WEIRD—non-Latinx White, highly educated, industrialized, rich, and democratic—populations (Apicella et al., 2020; Henrich et al., 2010). Moreover, while cross-cultural investigations of episodic memory are scarce, most of these existing studies perform “West vs. Rest” comparisons, grouping rich and diverse cultures into monoliths and leaving behind a vast majority of the population. Therefore, whereas our past experiences can shape what we remember, and culture is one such factor that may contribute to these effects, a lot of what we know about memory is based on biased samples. Conscious efforts to move away from convenience samples, and funding for underrepresented populations and diverse research teams are of extreme importance for our understanding of memory to truly be universal.

### Episodic memory over the lifespan: influence of aging

Episodic memories are highly prone to decline with age (Leal & Yassa, 2015). Memory deficits in aging have been associated with changes in synaptic plasticity in the hippocampus. For example, a selective vulnerability of the lateral entorhinal cortex (LEC)/PRC pathway has been reported in aging (Burke et al., 2012; Liu et al., 2008; Moyer et al., 2011), which largely supports the *what* stream in memory. Thus, there appears to be a more selective deficit of *what* memory compared with *where* memory in older age (Beaudet et al., 2015; Reagh & Yassa, 2014b; Reagh et al., 2014); where there is less activity for object stimuli in LEC, but no age differences on the medial EC stream for spatial stimuli (Reagh et al., 2016). The vulnerability of the EC in aging and AD could also explain some of the deficits in grid cell functioning, which lead to age-related navigational decline (Becker, 2022; Moser et al., 2015).

Additionally, across species, there is evidence of hippocampal hyperactivity in the DG/CA3 subregion of the hippocampus in old relative to young adults (Wilson et al., 2006; Yassa et al., 2011). This in addition to a reduction of perforant path input from the EC into the hippocampus drives the shift away from pattern separation and toward pattern completion with age, resulting in a greater impact of interference in the face of highly similar experiences (Leal & Yassa, 2018). Cross-species evidence supports the idea that aging is associated with a bias away from hippocampal pattern separation and recollection, and toward pattern completion and familiarity (Burke et al., 2010; Farovik et al., 2011; Westerberg et al., 2006; Yassa et al., 2011). While we typically view these changes in episodic memory as an impairment relative to young adult memory performance, it is possible that this shift toward generalization in aging is adaptive or appropriate as one ages. More work in this area needs to be done to determine why this shift occurs. There is lots of variability in memory performance with age (Leal et al., 2017), which could be due to underlying pathology, cognitive reserve, and many more factors that impact aging and memory. As we age, we have many more experiences and many more opportunities for interference in memory, which may influence why we remember what we remember as we get older (Leal & Yassa, 2014).

## Beyond theory: how to test and consider the *why*

Moving beyond theory, we want to briefly expand on important ways to explore the interaction between *what, when*, and *where*, while considering the *why*. There are several outstanding questions and gaps in the literature related to why we remember what we remember. Three important principles to consider are 1) using and developing laboratory tasks that test what we aim to test in the context of episodic memory in the real-world; 2) considering other factors that might play a role in impacting measures of memory, even if not the primary measure of interest; and 3) considering the neurobiological underpinnings of episodic memory more holistically.

Most standard laboratory-based episodic memory tasks do not consider or capture the nuance encompassed in episodic memory as we experience it in the real-world. There is always a trade-off between a well-controlled laboratory experiment, often focused on one specific element of episodic memory, and more naturalistic memory paradigms that bring with it more complexity and difficulty interpreting the driving factors of the results. We suggest that both types of experiments are essential for the richest understanding of episodic memory, where examining the whole is greater than the sum of its parts.

When considering study design, thinking carefully about what our paradigms can and cannot test is essential. If a study is designed to understand episodic memory, we must consider how the four *W*s may play a role in our experimental paradigms and whether the tasks that we use are tapping into how episodic memory might behave in the real world. If we only test one feature, we are limited to making claims about how we remember that feature in the more complex and dynamic real-world settings, in which the *what, where*, and *when* are almost always integrated and couched within the context of *why*. Episodic memory tasks, such as recalling a list of words or details of a specific event, are designed to manipulate prior events, making them ideal for experimental investigations into how memories are formed, maintained, and altered (Conway & Pleydell-Pearce, 2000). However, these methods lack insight into how people integrate their experiences into a coherent narrative of their lives (e.g., the autobiographical component of episodic memory).

It is crucial to note that performance on laboratory memory tasks does not always correspond to naturalistic memory abilities (McDermott et al., 2009). Creating ecologically valid memory paradigms is imperative for a more holistic understanding of memory function, as many traditional memory tasks do not account for additional barriers our memory system must face, such as differentiating between subtle changes in experiences from day to day. An example would be remembering faces and names and identifying them despite fine modifications, such as your sibiling rocking a new hair color, or correctly remembering that your coworkers name is María and not Mary. Despite this being a common struggle for many people, that only gets more difficult as we age, most traditional face-name associative memory tasks do not parametrically vary overlapping features of faces and names and often lack naturalistic facial features (e.g., hair, accessories, similarity of features, emotional expressions) (Mannion et al., 2023).

Thus, to enhance the authenticity of memory assessments, it is essential to create laboratory tasks that mirror natural encoding and retrieval processes and that capture the intricacies of everyday experiences, which play a role in what we remember. Findings are more likely to generalize to everyday life, providing insights into the practical functions and adaptive purposes of memory. By studying memory within a context that people naturally encounter, we gain a deeper understanding of the evolutionary and functional significance for why we remember, beyond the constraints of artificial laboratory settings.

Similarly, most laboratory measures are not generalizable to everyday environments. Recent efforts to use more naturalistic paradigms can help us to elucidate better about how memory functions in the real-world: e.g., the effort of moving toward video and movie stimuli that are more dynamic and multimodal than static images (Bainbridge & Baker, 2022; Leal et al., 2019; Reagh & Ranganath, 2023). The use of tasks that measure beyond oversimplified principles of memory, including tasks that include more naturalistic stimuli, such as people of diverse racial and ethnic makeups (Mannion et al., 2023), the use of technological advancements, such as virtual and augmented reality (Iriye & St. Jacques, 2021; Penaud et al., 2023), and the use of real-world neural recordings in freely moving participants (Topalovic et al., 2023) are essential toward tapping into episodic memory more holistically. Likewise, testing these paradigms in more diverse populations, including but not limited to diversity in terms of national origin, race and ethnicity, disability, age, sex and gender, education level, socioeconomic status, language, etc., and testing memory over extended time delays, will allow us to better comprehend typically occurring memory processes. Hence, using tasks that translate beyond a laboratory setting is crucial. Because episodic memory involves recalling personal experiences in specific contexts, encompassing not only the details of the event but also the circumstances surrounding the event and the reasons behind it, using ecologically valid tasks ensures that the assessment of memory resembles real-life situations more closely, improving relevance and applicability of the findings.

Another important element to consider when measuring memory performance are individual differences that we have discussed in this review. While this variability can be difficult to explore as it can fluctuate even within individuals, being aware of these potential drivers of performance in memory studies is a worthwhile effort to undertake to make findings more generalizable and reproducible. Consciously moving away from convenience samples, fostering diverse research groups, and obtaining qualitative information from participants can help to ameliorate some of these issues, where a better understanding of these elements can provide additional clues into the *why.*

In addition, most studies have highlighted that the PRC and PHC receive *what* and *where* inputs, respectively, to later converge in the hippocampus to create multimodal, rich memories. However, the literature exploring whether a *when* and a *why* stream exist is extremely limited. We attempt to create a more holistic view of these processes (Fig. 1) that includes all four *W*s in the context of the MTL but also the rest of the brain that is highly involved in episodic memory processing. Whereas the *when* and w*hy* streams may be more complex and difficult to study, examining patterns of activation within MTL and cortical areas during encoding and retrieval with these domains in mind will be important for future studies to consider. Perhaps there is more than one *why *stream that brings differing relevant *why* information (e.g., reward, attention, emotion) to the MTL to assist the memory processing and prioritization.

## Conclusions

Throughout this review, we synthesized past and current knowledge to unravel the complex tapestry of episodic memory, emphasizing the importance of understanding *why* we remember. By integrating findings from neuroscience, psychology, and related disciplines, we aimed to provide a comprehensive framework to those seeking a deeper understanding of episodic memory in the real world. This exploration not only advances our theoretical understanding but also has practical implications for memory-related interventions and educational practices.

Expanding our perspective of episodic memory and considering memory as taking place within an individual with a unique set of past experiences with certain goals, motivations, and internal states is essential to understanding real-world episodic memory. This holistic view of episodic memory could provide unique insight into how memory works, which could shape how we think about and develop interventions for memory impairment. We must embrace the complexity of episodic memory if we truly want to understand how it works. It is clear that the field is seeking this expansion given the increasing number of studies developing more naturalistic memory tasks and widening and diversifying the study of individuals, considering culture, age, race/ethnicity, sex/gender, etc., as important drivers of memory performance that have gone understudied. Although there are clear gaps in the literature, both neurobiological and behavioral, efforts to bridge our current knowledge with future work are necessary to lay the groundwork for new studies to elucidate *why* we remember and forget the *what, where*, and *when*.

## Data Availability

Not applicable.
